# Noninvasive Evaluation of Metabolic Tumor Volume in Lewis Lung Carcinoma Tumor-Bearing C57BL/6 Mice with Micro-PET and the Radiotracers ^18^F-Alfatide and ^18^F-FDG: A Comparative Analysis

**DOI:** 10.1371/journal.pone.0136195

**Published:** 2015-09-09

**Authors:** Yu-Chun Wei, Xudong Hu, Yongsheng Gao, Zheng Fu, Wei Zhao, Qingxi Yu, Suzhen Wang, Shouhui Zhu, Jun Li, Jinming Yu, Shuanghu Yuan

**Affiliations:** 1 Department of Radiation Oncology, Shandong Cancer Hospital and Institute, Jinan, China; 2 School of Medicine and Life Sciences, University of Jinan-Shandong Academy of Medical Sciences, Jinan, China; 3 Department of Pathology, Shandong Cancer Hospital and Institute, Jinan, China; 4 Department of Nuclear Medicine, Shandong Cancer Hospital and Institute, Jinan, China; 5 Department of Thoracic Surgery, Shandong Province Hospital, Jinan, China; Van Andel Institute, UNITED STATES

## Abstract

**Purpose:**

To explore the value of a new simple lyophilized kit for labeling PRGD_2_ peptide (^18^F-ALF-NOTA-PRGD_2_, denoted as ^18^F-alfatide) in the determination of metabolic tumor volume (MTV) with micro-PET in lewis lung carcinoma (LLC) tumor-bearing C57BL/6 mice verified by pathologic examination and compared with those using ^18^F-fluorodeoxyglucose (FDG) PET.

**Methods:**

All LLC tumor-bearing C57BL/6 mice underwent two attenuation-corrected whole-body micro-PET scans with the radiotracers ^18^F-alfatide and ^18^F-FDG within two days. ^18^F-alfatide metabolic tumor volume (V_RGD_) and ^18^F-FDG metabolic tumor volume (V_FDG_) were manually delineated slice by slice on PET images. Pathologic tumor volume (V_Path_) was measured in vitro after the xenografts were removed.

**Results:**

A total of 37 mice with NSCLC xenografts were enrolled and 33 of them underwent ^18^F-alfatide PET, and 35 of them underwent ^18^F-FDG PET and all underwent pathological examination. The mean ± standard deviation of V_Path_, V_RGD,_ and V_FDG_ were 0.59±0.32 cm^3^ (range,0.13~1.64 cm^3^), 0.61±0.37 cm^3^ (range,0.15~1.86 cm^3^), and 1.24±0.53 cm^3^ (range,0.17~2.20 cm^3^), respectively. V_Path_ vs. V_RGD_, V_Path_ vs. V_FDG_, and V_RGD_ vs. V_FDG_ comparisons were t = -0.145, *P* = 0.885, t = -6.239, *P*<0.001, and t = -5.661, *P*<0.001, respectively. No significant difference was found between V_Path_ and V_RGD_. V_FDG_ was much larger than V_RGD_ and V_Path_. V_RGD_ seemed more approximate to the pathologic gross tumor volume. Furthermore, V_Path_ was more strongly correlated with V_RGD_ (R = 0.964,*P*<0.001) than with V_FDG_ (R = 0.584,*P*<0.001).

**Conclusions:**

^18^F-alfatide PET provided a better estimation of gross tumor volume than ^18^F-FDG PET in LLC tumor-bearing C57BL/6 mice.

## Introduction

Improving the accuracy of target volume estimation will help avoid unnecessary radiation of normal tissues and help avoid geographic tumor misses in patients with non-small cell lung cancer (NSCLC). Accurate metabolic tumor volume (MTV) assessment is a promising method for defining volumes in radiotherapy because of the development of functional imaging tools and image-guided radiotherapy.

PET with ^18^F-FDG has been widely used to define target volumes in radiotherapy with increased metabolism. Studies have shown that FDG PET/CT can reduce inter- and intra-observer variability of target volume delineation to improve radiotherapy treatment planning [1, 2]. In recent years, the possibly substantial impact of ^18^F-FDG PET on the size and form of target volumes in lung cancer was demonstrated [3]. Most methods currently used in clinical practice are based on the use of some form of binary threshold, either fixed [4] or adaptive, that use tumor-to-background ratios [5]. Unfortunately, sometimes ^18^F-FGD PET fail to provide satisfactory delineation of tumors characterized by heterogeneous activity distributions and fail to provide reproducible results for small tumors with low contrast because of intense cardiac uptake and high lung background [6]. Realistically, it is preferable that the radiotherapist uses the ^18^F-FDG PET images as a reference only [7]. The radiation oncologist are expecting more accurate tool to contour target volume precisely.

Arginine-glycine-aspartic acid peptide (Arg-Gly-Asp, RGD) can specifically bind with integrin αvβ3, which is highly expressed in angiogenic tumors, to detect angiogenesis in non-invasive PET imaging. Angiogenesis plays an important role in the regulation of tumor growth, local invasiveness, and metastatic potential [8]. Chen et al invented a new simple lyophilized kit for labeling PRGD_2_ peptide (^18^F-ALF-NOTA-PRGD_2_, denoted as ^18^F-alfatide) [9]. PET scanning with RGD allows specific imaging of integrin αvβ3 expression with minimal nonspecific activity accumulation in normal lung and heart tissue. Therefore, RGD PET may render high-quality orthotopic lung cancer images, enabling clear demarcation of both the primary tumor at the upper lobe of the left lung, as well as metastases in the mediastinum, contralateral lung, and diaphragm.

This study is designed to explore the value of ^18^F-alfatide in the determination of MTV with micro-PET in lewis lung carcinoma (LLC) tumor-bearing C57BL/6 mice verified by pathologic examination and compared with those using ^18^F-FDG PET.

## Materials and Methods

### Cell Culture and Animal Tumor Model Preparation

Murine LLC cells, recently used in a number of high-profile preclinical studies [10,11], was purchased from the Type Culture Collection of the Chinese Academy of Sciences, Shanghai, China. Murine LLC cells were grown in RPMI 1640, supplemented with 10% fetal bovine serum and 1% penicillin streptomycin antibiotic mixture in a humidified incubator (Heraeus, Hanau, Germany) at 37°C with 5% CO_2_ atmosphere. LLC was injected (2.5×10^5^ cells/100 μl/mouse) into the right hind leg muscle of C57BL/6 mice.

Thirty-seven C57BL/6 inbred male mice were housed in a limited access animal facility. Animal room temperature and relative humidity were set at 22±2°C and 55±10%, respectively. Artificial lighting provided a 24 h cycle of 12 h light/12 h dark (7 a.m. to 7 p.m.). All animal procedures were in accordance with the Shandong Cancer Hospital & Institute Ethical Committee Guide for the care and use of Laboratory Animals. The Shandong Cancer Hospital & Institute Ethical Committee specifically approved this study.

### ^18^F-alfatide PET and ^18^F-FDG PET image acquisition

All mice underwent PET scans (Siemens Medical Solutions) using ^18^F-alfatide and ^18^F-FDG PET respectively within 2 days when tumor diameter reached approximately 10 mm. With the assistance of the Inveon system’s positioning laser, the LLC tumor-bearing mouse was placed with its tumor located at the center of the field of view (FOV), where the highest imaging sensitivity can be achieved. ^18^F-alfatide PET scans were performed 60 minutes after tail-vein injection of 2.4–3.5 MBp of ^18^F-Alfatide under isoflurane anesthesia. During the acquisition period, a thermostat-controlled thermal heater maintained the body temperature of the mice. ^18^F-FDG PET scans were performed for alll mice after 4 hours fasting and 60 minutes after injection of ^18^F-FDG at a dosage of 2.6–3.6 MBp. The mice rested quietly in a warm box under isoflurane anesthesia for approximately 1 hour. Subsequently, the mice underwent the scanning, using the same parameters that had been employed for the ^18^F-Alfatide PET scan.

The images were reconstructed and analyzed using Inveon Acquisition Workplace v1.4.3 SP1 software. In the bio-distribution analysis of static images, regions of interest (ROIs) were placed over the tumor. The attenuation-corrected PET images were reconstructed and reviewed in axial, coronal, and sagittal planes; the same procedure was performed with a cine display of maximum-intensity projections of the PET data.

**Fig 1:**
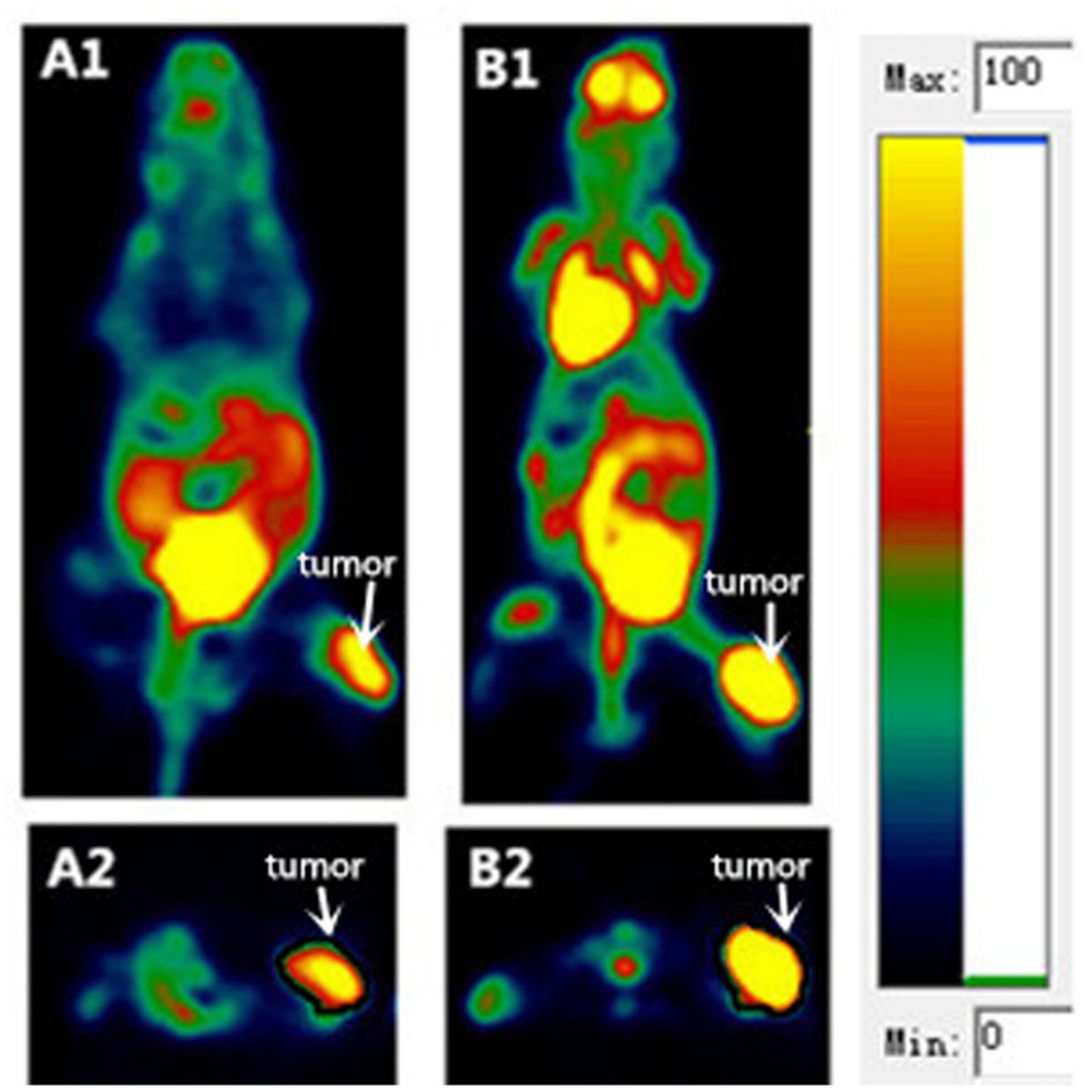
PET imaging. PET images showing localization of ^18^F-alfatide (**A1**, coronal; **A2**, transverse) and ^18^F-FDG (**B1**, coronal; **B2** transverse) in the same mice with lung cancer xenografts. See the color bar for PET images.

The ^18^F-alfatide metabolic tumor volume (V_RGD_) and ^18^F-FDG metabolic tumor volume (V_FDG_) were manually delineated slice by slice on the ^18^F-alfatide and ^18^F-FDG PET images (Fig 1). As a first step, an experienced physician (Z.F) used the ROI standard evaluation tool provided by the manufacture of the micro-PET system and a global logarithmic scaling to generate a “visual” PET GTV, comprising the tissue considered visually as part of the malignant primary tumor. ROI was positioned around the tumors slice by slice and obtained a set of data such as max (ROImax), mean and so on. The results were expressed as the standardized uptake value (SUVmax), which was calculated according to the following formula: ROImax×9500(CF value)×body weight [g]/injected activity [Bq]). The MTV was delineated on the PET images in transaction slice by slice with the 40% of the SUVmax, a threshold that has been used for the delineation tumor volume in previously study [12].

### Pathologic volume measurement

**Fig 2:**
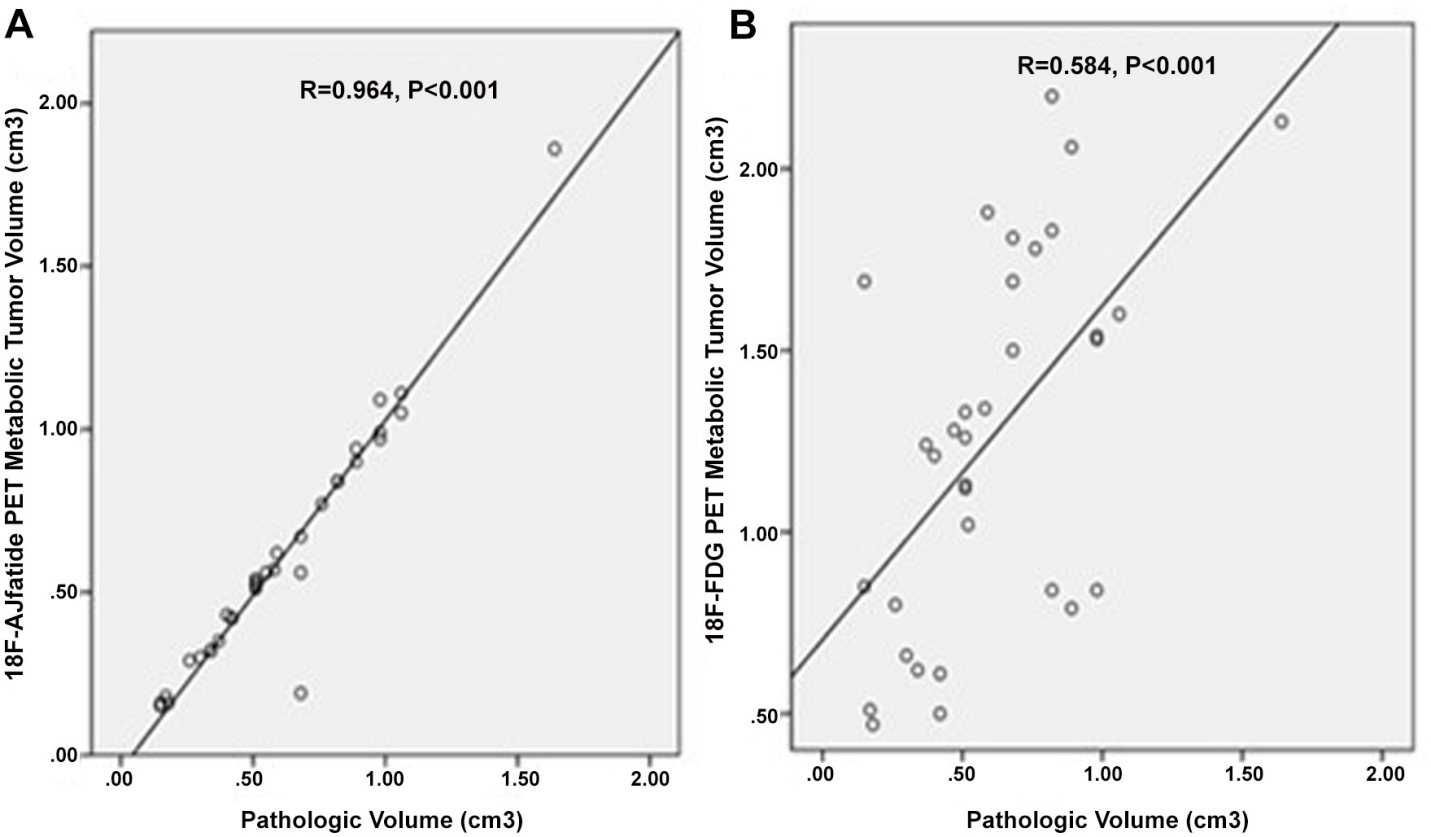
Correlation between ^18^F-alfatide metabolic tumor volume (V_RGD_) and ^18^F-FDG PET metabolic tumor volume (V_FDG_) and pathologic volume(V_Path_). A strong significant correlation was found between V_RGD_ and V_Path_ (**A**, R = 0.964, *P*<0.001). A moderately significant correlation was found between V_FDG_ and V_Path_ (**B**, R = 0.584, *P*<0.001).

All mice underwent tumor resection after the end of imaging (Fig 2). Specimens that were submitted fresh from the operating laboratory had three dimensional gross measurements taken from the tumor before being placed into 10% formalin. The pathologic tumor volume (V_Path_) was estimated from the volume of an ellipsoid:

$${\text{V}}_{\text{Path}}=\pi /6\times {\text{D}}_{\text{long}}\times {\text{D}}_{\text{short}}{}^{2}.$$

where D_long_ and D_short_ were the longest and shortest diameters on the transverse plane before formalin fixation. D_long_ and D_short_ were the two orthogonal diameters obtained from the resected tumor specimen.

### Statistical analysis

Statistical analysis was performed using SPSS software (version 17.0; SPSS, Inc). Student’s unpaired t-test was used to detect differences between two sample means. The relationships between V_Path_ and V_RGD_ and between V_Path_ and V_FDG_ were tested by the Linear Regression Equation. All analyses were 2-sided, and a *P* value of less than 0.05 was considered statistically significant.

## Results

A total of 37 mice with NSCLC xenografts underwent pathologic examination after completing the PET imaging. Each mouse underwent ^18^F-alfatide and ^18^F-FDG PET imaging. In the ^18^F-alfatide PET imaging process, 4 mice were not scanned because of intravenous administration failure. For the same reason, 2 mice did not undergo ^18^F-FDG PET scanning.

Tumor volume was measured by various methods (Table 1). The MTV was delineated in PET imaging performance of a novel αVβ3 integrin radiotracer, denoted as ^18^F-alfatide, and ^18^F-FDG. V_RGD_ (n = 33) was significantly smaller than V_FDG_ (n = 35), 0.61±0.37 vs. 1.24±0.53 cm^3^, t = -5.661, *P*<0.001. This finding is illustrated by Fig 1-A1 and 1-A2 and Fig 1-B1 and 1-B2, where length×width of the xenograft on ^18^F-FDG PET images were larger than ^18^F-alfatide PET images.

**Table 1:** Tumor size, actual pathologic volume, ^18^F-alfatide metabolic volume, and ^18^F-FDG metabolic volume for each case.

NO	D_long_(cm)	D_short_(cm)	V_path_(cc)	V_RGD_(cc)	V_FDG_(cc)
1	1.00	0.70	0.26	0.29	0.80
2	1.20	0.90	0.51	0.51	1.13
3	1.30	1.20	0.98	0.97	0.84
4	1.60	1.40	1.64	1.86	2.13
5	1.40	1.20	1.06	1.11	1.60
6	1.40	1.20	1.06	1.05	——
7	1.30	1.00	0.68	0.56	1.81
8	1.00	0.90	0.42	0.42	0.61
9	1.00	1.00	0.52	——	1.02
10	1.20	0.90	0.51	0.53	1.26
11	1.30	1.00	0.68	0.19	1.69
12	1.30	1.10	0.82	0.84	0.84
13	1.10	1.00	0.58	0.57	1.34
14	1.30	0.90	0.55	0.56	——
15	1.20	0.90	0.51	0.54	1.33
16	0.80	0.60	0.15	0.15	1.69
17	1.40	0.90	0.59	0.62	1.88
18	1.30	1.00	0.68	0.67	1.50
19	1.00	0.80	0.34	0.32	0.62
20	1.10	0.90	0.47	——	1.28
21	1.40	1.10	0.89	0.94	2.06
22	1.10	0.80	0.37	0.35	1.24
23	0.90	0.60	0.17	0.18	0.51
24	1.20	0.80	0.40	0.43	1.21
25	1.20	1.10	0.76	0.77	1.78
26	1.30	1.10	0.82	0.84	2.20
27	1.30	1.20	0.98	1.09	1.53
28	1.30	1.20	0.98	0.99	1.54
29	1.30	1.10	0.82	——	1.83
30	1.20	0.90	0.51	0.52	1.12
31	1.40	1.10	0.89	0.90	0.79
32	0.80	0.60	0.15	0.16	0.85
33	0.70	0.70	0.18	0.16	0.47
34	1.00	0.90	0.42	0.42	0.50
35	0.90	0.80	0.30	0.30	0.66
36	1.00	0.80	0.34	0.32	1.68
37	0.70	0.60	0.13	——	0.17

Dlong = long diameter on transverse plane; Dshort = short diameter on transverse plane; Vpath = pathologic volume; VRGD = 18F-alfatide metabolic tumor volume; VFDG = 18F-FDG metabolic tumor volume.

Table 2 shows the comparison of V_Path_ (n = 37) with V_RGD_ and V_FDG_. V_Path_ was considered the gold standard for MTV as it provides the closest estimation of real tumor volume. No difference was found between V_Path_ and V_RGD_ (0.59±0.32 vs. 0.61±0.37, t = -0.145, *P* = 0.885). V_Path_ was significantly smaller than V_FDG_ (0.59±0.32 vs. 1.24±0.53 cm^3^, t = -6.24, *P*<0.001).

**Table 2:** Pathologic Volume compared with ^18^F-alfatide and ^18^F-FDG PET MTV in LLC tumor-bearing C57BL/6 mice.

Group	Median (cm^3^)	Mean±SD (cm^3^)	Mean difference	95%CI difference	*t*	*P*
Vp_ath_	0.52	0.59±0.32	——	——	——	——
V_RGD_	0.54	0.61±0.37	-0.01±0.10	-0.21±0.19	-0.145	0.885
V_FDG_	1.26	1.24±0.53	-0.64±0.09	-0.84±0.44	-6.24	<0.001

MTV = metabolic tumor volume; Vp_ath_ = pathologic volume; V_RGD_ = ^18^F-alfatide metabolic tumor volume; V_FDG_ = ^18^F-FDG metabolic tumor volume.

The relationship between MTV and V_Path_ (Fig 2) was also analyzed. V_Path_ was strongly correlated with V_RGD_ (n = 33, R = 0.964, *P*<0.001). V_Path_ also had a moderately positive correlation with V_FDG_ (n = 35, R = 0.584, *P*<0.001). The change in MTV of various specimens measured by ^18^F-alfatide and ^18^F-FDG PET imaging was consistent with the actual tumor volume. However, ^18^F-alfatide PET more accurately reflected the actual tumor size than ^18^F-FDG PET.

## Discussion

The present study showed that larger MTVs were obtained with ^18^F-FDG PET imaging than ^18^F-alfatide PET imaging, and V_RGD_ was similar to V_Path_ in a mice model with NSCLC.

A study showed similar results in which the average tumor volume shown by ^18^F-FDG PET imaging at 6 hours (0.37±0.08 cm^3^), 24 hours (0.29±0.07cm^3^), and 48 hours (0.18±0.03 cm^3^) was larger than that estimated by histology (0.16±0.05cm^3^), (0.17±0.06 cm^3^), (0.06±0.02 cm^3^) [13]. In ^18^F-FDG PET imaging, many normal tissues display high uptake of ^18^F-FDG [14], and this process is modulated by disease states such as diabetes [15] or physical exertion before and during scanning. These factors complicate the interpretation of ^18^F-FDG PET imaging and help motivate the development of novel imaging tracers, such as ^18^F-alfatide in this present study. In contrast to V_FDG_, we did not find any prior studies evaluating the measurement of MTV by ^18^F-alfatide PET. ^18^F-FDG uptake is significantly correlated with glucose metabolism, but it is phosphorylated and trapped within cells rather than metabolized and is rapidly cleared from the bloodstream [16]. Tumors are metabolically active but many normal tissues also display high uptake of ^18^F-FDG. Para-tumor inflammation and infection may lead to high ^18^F-FDG uptake, which potentially confounds cancer imaging. Kyoichi Kaira [17] et al showed that in tumor tissues, the amount of FDG uptake is not only associated with molecules relevant to glucose metabolism but also has a close relationship with hypoxia, angiogenesis and the mTOR signaling pathway. Research has reported that the amount of FDG uptake is determined by the expression of glucose transporter-1 (GLUT1), hypoxia-inducible factor-1α (HIF-1α), vascular endothelial growth factor (VEGF), and microvessel density (MVD) [18, 19]. These factors may explain why the MTV of ^18^F-FDG PET is larger than V_Path_. Integrin αVβ3 is a member of the integrins family, and it can bind to a variety of plasma and extracellular matrix proteins containing the conserved amino acid sequence RGD [20]. The integrin αVβ3 receptor was expressed preferentially on various tumor cells and endothelial cells but was low on mature endothelial and epithelial cells [21, 22]. Our findings indicate that RGD has high affinity for NSCLC in this study. However, the mechanism underlying why the MTV of ^18^F-alfatide PET is closer to actual tumor volume is still unclear. Additionally, ^18^F-alfatide PET is still not a perfect tool for tumor contouring due to the heterogeneity of the malignant lesions and partial volume effect. The criteria used to define MTV are subjective, and defining MTV depends on the interpretation of appropriate parameter settings in the evaluation of the image and resulting target volume contours [23]. The tumors had to be imaged with two tracers sequentially within two days as opposed to simultaneously, and therefore, variations in growth patterns could have partially influenced the results. Another limitation is that the LLC tumor model involves implantation of a tumor into a known location, and therefore, observers were aware of the tumor location. Furthermore, clinical situations are far more complicated than the animal models. Whether ^18^F-alfatide PET has additional value than ^18^F-FDG PET on tumor volume contouring is still unknown in patients with NSCLC and it deserves further study.

## Conclusions

^18^F-alfatide PET provided a better estimation of gross tumor volume than ^18^F-FDG PET in LLC tumor-bearing C57BL/6 mice.

## Supporting Information

S1 ARRIVE Guidelines Checklist NC3Rs ARRIVE Guidelines Checklist. (DOCX)
