# Supplementary material for: Noninvasive Evaluation of Metabolic Tumor Volume in Lewis Lung Carcinoma Tumor-Bearing C57BL/6 Mice with Micro-PET and the Radiotracers 18F-Alfatide and 18F-FDG: A Comparative Analysis
Source: PLoS One. 2015 Sep 9;10(9):e0136195. doi: 10.1371/journal.pone.0136195 (PMC4564167; doi:10.1371/journal.pone.0136195)
Supplement: S1 ARRIVE Guidelines Checklist — (DOCX) [file pone.0136195.s001.docx]

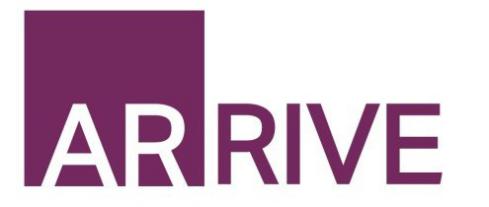


The ARRIVE Guidelines Checklist

Animal Research: Reporting In Vivo Experiments

Carol Kilkenny1, William J Browne2, Innes C Cuthill3, Michael Emerson4 and Douglas G Altman5

*1The National Centre for the Replacement, Refinement and Reduction of Animals in Research, London, UK, 2School of Veterinary Science, University of Bristol, Bristol, UK, 3School of Biological Sciences, University of Bristol, Bristol, UK, 4National Heart and Lung Institute, Imperial College London, UK, 5Centre for Statistics in Medicine, University of Oxford, Oxford, UK.*

|  | ITEM | RECOMMENDATION | | Section/ Paragraph | |
| --- | --- | --- | --- | --- | --- |
| Title 1 Provide as accurate and concise a description of the content of the article as possible. | | | Page 9 | |  |
| Abstract 2 Provide an accurate summary of the background, research objectives, including details of the species or strain of animal used, key methods, principal findings and conclusions of the study. | | | Page 10 Abstract | |  |
| INTRODUCTION | | |  | |  |
| Background 3 a. Include sufficient scientific background (including relevant references to previous work) to understand the motivation and context for the study, and explain the experimental approach and rationale.  b. Explain how and why the animal species and model being used can address the scientific objectives and, where appropriate, the study’s relevance to human biology. | | | Page 11 Introduction | |  |
| Objectives 4 Clearly describe the primary and any secondary objectives of the study, or specific hypotheses being tested. | | | Page 12 Introduction | |  |
| METHODS | | |  | |  |
| Ethical statement 5 Indicate the nature of the ethical review permissions, relevant licences (e.g.  Animal [Scientific Procedures] Act 1986), and national or institutional  guidelines for the care and use of animals, that cover the research. | | | Page 12 Methods and Materials | |  |
| Study design 6 For each experiment, give brief details of the study design including:  a. The number of experimental and control groups.  b. Any steps taken to minimise the effects of subjective bias when allocating animals to treatment (e.g. randomisation procedure) and when assessing results (e.g. if done, describe who was blinded and when).  c. The experimental unit (e.g. a single animal, group or cage of animals).  A time-line diagram or flow chart can be useful to illustrate how complex study designs were carried out. | | | Page 12 | |  |
| Experimental 7 For each experiment and each experimental group, including controls,  procedures provide precise details of all procedures carried out. For example:  a. How (e.g. drug formulation and dose, site and route of administration, anaesthesia and analgesia used [including monitoring], surgical procedure, method of euthanasia). Provide details of any specialist equipment used, including supplier(s).  b. When (e.g. time of day).  c. Where (e.g. home cage, laboratory, water maze).  d. Why (e.g. rationale for choice of specific anaesthetic, route of administration, drug dose used). | | | Page 13 PET image acquisition | |  |
| Experimental 8 a. Provide details of the animals used, including species, strain, sex,  animals developmental stage (e.g. mean or median age plus age range) and weight (e.g. mean or median weight plus weight range).  b. Provide further relevant information such as the source of animals, international strain nomenclature, genetic modification status (e.g. knock-out or transgenic), genotype, health/immune status, drug or test naïve, previous procedures, etc. | | | Page 12 Methods and Materials | |  |

The ARRIVE guidelines. Originally published in *PLoS Biology*, June 20101

| Housing and 9 Provide details of:  husbandry a. Housing (type of facility e.g. specific pathogen free [SPF]; type of cage or housing; bedding material; number of cage companions; tank shape and material etc. for fish).  b. Husbandry conditions (e.g. breeding programme, light/dark cycle, temperature, quality of water etc for fish, type of food, access to food and water, environmental enrichment).  c. Welfare-related assessments and interventions that were carried out prior to, during, or after the experiment. | Page 12 Methods and Materials |
| --- | --- |
| Sample size 10 a. Specify the total number of animals used in each experiment, and the number of animals in each experimental group.  b. Explain how the number of animals was arrived at. Provide details of any sample size calculation used.  c. Indicate the number of independent replications of each experiment, if relevant. | Page 12 Methods and Materials |
| Allocating 11 a. Give full details of how animals were allocated to experimental groups,  animals to including randomisation or matching if done.  experimental b. Describe the order in which the animals in the different experimental  groups groups were treated and assessed. | Page 13 Methods and Materials |
| Experimental 12 Clearly define the primary and secondary experimental outcomes assessed  outcomes (e.g. cell death, molecular markers, behavioural changes). | Page 16 Results |
| Statistical 13 a. Provide details of the statistical methods used for each analysis.  methods b. Specify the unit of analysis for each dataset (e.g. single animal, group of animals, single neuron).  c. Describe any methods used to assess whether the data met the assumptions of the statistical approach. | Page 15 Statistical analysis |
| RESULTS |  |
| Baseline data 14 For each experimental group, report relevant characteristics and health status of animals (e.g. weight, microbiological status, and drug or test naïve) prior to treatment or testing. (This information can often be tabulated). | Page 16 Results |
| Numbers 15 a. Report the number of animals in each group included in each analysis.  analysed Report absolute numbers (e.g. 10/20, not 50%2).  b. If any animals or data were not included in the analysis, explain why. | Page 16 Results |
| Outcomes and 16 Report the results for each analysis carried out, with a measure of precision  estimation (e.g. standard error or confidence interval). | Page 16 Results |
| Adverse events 17 a. Give details of all important adverse events in each experimental group. b. Describe any modifications to the experimental protocols made to  reduce adverse events. | —— |
| DISCUSSION |  |
| Interpretation/ 18 a. Interpret the results, taking into account the study objectives and scientific hypotheses, current theory and other relevant studies in the literature. implications b. Comment on the study limitations including any potential sources of bias,  any limitations of the animal model, and the imprecision associated with the results2.  c. Describe any implications of your experimental methods or findings for the replacement, refinement or reduction (the 3Rs) of the use of animals in research. | Page 18 Discussion |
| Generalisability/ 19 Comment on whether, and how, the findings of this study are likely to  translation translate to other species or systems, including any relevance to human biology. | —— |
| Funding 20 List all funding sources (including grant number) and the role of the funder(s) in the study. | —— |

References:


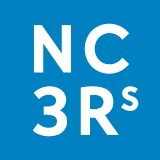


1. Kilkenny C, Browne WJ, Cuthill IC, Emerson M, Altman DG (2010) Improving Bioscience Research Reporting: The ARRIVE Guidelines for Reporting Animal Research. *PLoS Biol* 8(6): e1000412. doi:10.1371/journal.pbio.1000412

2. Schulz KF, Altman DG, Moher D, the CONSORT Group (2010) CONSORT 2010 Statement: updated guidelines for reporting parallel group randomised trials. *BMJ* 340:c332.
